# A Typology of Social Media Use by Human Service Nonprofits: Mixed Methods Study

**DOI:** 10.2196/51698

**Published:** 2024-05-08

**Authors:** Jia Xue, Micheal L Shier, Junxiang Chen, Yirun Wang, Chengda Zheng, Chen Chen

**Affiliations:** 1 Factor-Inwentash Faculty of Social Work University of Toronto Toronto, ON Canada; 2 Faculty of Information University of Toronto Toronto, ON Canada; 3 Department of Biostatistics and Health Data Science Indiana University School of Medicine Indianapolis, IN United States; 4 Artificial Intelligence for Justice Lab University of Toronto Toronto, ON Canada

**Keywords:** human service nonprofits, sexual assault support centers, Canada, typology, theory, Twitter, machine learning, social media, tweet, tweets, nonprofit, nonprofits, crisis, sexual assault, sexual violence, sexual abuse, support center, support centers, communication, communications, organization, organizations, organizational, sentiment analysis, business, marketing

## Abstract

**Background:**

Nonprofit organizations are increasingly using social media to improve their communication strategies with the broader population. However, within the domain of human service nonprofits, there is hesitancy to fully use social media tools, and there is limited scope among organizational personnel in applying their potential beyond self-promotion and service advertisement. There is a pressing need for greater conceptual clarity to support education and training on the varied reasons for using social media to increase organizational outcomes.

**Objective:**

This study leverages the potential of Twitter (subsequently rebranded as X [X Corp]) to examine the online communication content within a sample (n=133) of nonprofit sexual assault (SA) centers in Canada. To achieve this, we developed a typology using a qualitative and supervised machine learning model for the automatic classification of tweets posted by these centers.

**Methods:**

Using a mixed methods approach that combines machine learning and qualitative analysis, we manually coded 10,809 tweets from 133 SA centers in Canada, spanning the period from March 2009 to March 2023. These manually labeled tweets were used as the training data set for the supervised machine learning process, which allowed us to classify 286,551 organizational tweets. The classification model based on supervised machine learning yielded satisfactory results, prompting the use of unsupervised machine learning to classify the topics within each thematic category and identify latent topics. The qualitative thematic analysis, in combination with topic modeling, provided a contextual understanding of each theme. Sentiment analysis was conducted to reveal the emotions conveyed in the tweets. We conducted validation of the model with 2 independent data sets.

**Results:**

Manual annotation of 10,809 tweets identified seven thematic categories: (1) community engagement, (2) organization administration, (3) public awareness, (4) political advocacy, (5) support for others, (6) partnerships, and (7) appreciation. Organization administration was the most frequent segment, and political advocacy and partnerships were the smallest segments. The supervised machine learning model achieved an accuracy of 63.4% in classifying tweets. The sentiment analysis revealed a prevalence of neutral sentiment across all categories. The emotion analysis indicated that fear was predominant, whereas joy was associated with the partnership and appreciation tweets. Topic modeling identified distinct themes within each category, providing valuable insights into the prevalent discussions surrounding SA and related issues.

**Conclusions:**

This research contributes an original theoretical model that sheds light on how human service nonprofits use social media to achieve their online organizational communication objectives across 7 thematic categories. The study advances our comprehension of social media use by nonprofits, presenting a comprehensive typology that captures the diverse communication objectives and contents of these organizations, which provide content to expand training and education for nonprofit leaders to connect and engage with the public, policy experts, other organizations, and potential service users.

## Introduction

### Background

It has long been acknowledged that social media plays a significant role in facilitating stakeholder engagement between nonprofits and community members [1-3]. Human service nonprofits have recognized the potential of social media in securing donations; recruiting volunteers [4-7]; enhancing trust, accountability, and awareness [8]; and fostering partnerships [9]. However, research on the specific focus of social media engagement by human service nonprofits remains somewhat limited in the existing literature and practice [10]. Traditionally, social media in the nonprofit sector has been extensively explored in relation to its use for advocacy purposes [11-13]. Although investigation into the advocacy function of social media use is important within the human service nonprofit sector, as this is a key role played by human service nonprofits to promote and support social welfare development, the need of these organizations to engage with the wider community is much more expansive.

Human service organizations are complex entities involved in a wide range of activities to fulfill their missions, primarily focused on providing direct support to address negative social, economic, and political outcomes of social groups considered marginalized and to promote social welfare development through program development, public awareness, and advocacy efforts [14]. These human service nonprofits interact with diverse human resources (professional and volunteer), service users, and community groups; form partnerships across sectors (nonprofits, for-profit firms, and governments); and manage activities with for-profit (eg, social enterprises, social investors, and consumers), government (eg, contracting arrangements), and nonprofit (eg, through foundations) revenue sources and organizations. The use of social media is complicated further when considering its use for service user engagement, as there is an emerging body of literature on the use of social media for service delivery–related purposes [15,16].

To move beyond the advocacy-related function of social media by human service nonprofits, this research investigated in greater detail the various reasons why human service nonprofits are using social media within the sexual violence service delivery sector across Canada. The research merges social science, big data, and computer science to further enhance our knowledge and understanding of how human service nonprofit organizations use information communication technology. This study expands upon prior studies of nonprofit organizational communication research by using Twitter-based data (subsequently rebranded as X [X Corp]). Human-labeled tweets were used as training data, and a supervised machine learning approach was used to automatically predict content analytical themes in a Twitter corpus. The study builds a predictive classification model that uses a supervised machine learning algorithm to evaluate large social media data sets, resulting in a theoretical framework that categorizes the objectives of the sexual assault (SA) organization posts on social media. The overarching question that guides this research is as follows: “What are the different purposes of social media communication among SA centers in Canada?” This research is part of a greater effort to develop a strategic approach and educational information for training human service personnel and leaders on the use of social media to increase the capacity of human service nonprofits [17,18].

### Literature Review

Current research indicates that nonprofit organizations are increasingly using social media to improve their communication strategies with the broader population. A primary focus of research in this area has been on the specific tangible ways of this type of engagement, including the volume of engagement and the focus of messaging, along with its directionality, and the emphasis of the posts being informative and practical [19-21]. For example, Guo and Saxton [22] have focused on the extent to which nonprofits are gaining attention and highlight that this is influenced by the size of an organization’s network, the frequency with which it communicates through social media, and the number of conversations an organization joins [22]. This research is important, as it highlights the mechanisms of social media use and the frequency; however, it does not provide sufficient insight into the various reasons for social media use and the outcomes of this communication strategy on different organizational functions or purposes, and particularly important within the realm of human service nonprofit organization, which may use social media to achieve a multitude of objectives.

In fact, research on social media use within human service nonprofits specifically has identified some hesitancy to use social media education or useful tools to focus on social media use [23,24], and there is limited scope among organizational personnel in applying its usefulness beyond promoting one’s organization and its services [25]. This lack of engagement has been determined to be influenced in part due to limited education and awareness of the utility of social media use in the human services sector and other key organizational dynamics such as organizational culture, funding, and size of the organization [6,24,26,27].

Furthermore, a strong focus within the literature has been on how social media has been impacted by market actors (such as donors), which has constrained the framing of social media messaging [20,28-30]. Likewise, challenges with social media use, such as breaches of confidentiality and its increased use for surveillance and accountability-related purposes [31], also act to constrain social media use. As a result, there is a need for greater conceptual clarity to support education and training on the varied reasons for using social media to increase organizational outcomes [32-34].

This research seeks to address these gaps by investigating the wider range of social media use by human service nonprofits, establishing a typology of reasons for social media use beyond advocacy-related purposes. By doing so, it also addresses concerns regarding limited education and training within the sector on leveraging social media for diverse organizational objectives. Through the incorporation of machine learning and content analysis, this study contributes to a deeper understanding of nonprofit communication strategies and offers practical implications for improved social media engagement within the human service context.

### Aim of the Study

This study investigates the objectives of social media engagement and the contents posted by human service nonprofit organizations on the social media platform Twitter, with a particular focus on SA service delivery centers in Canada. To achieve this aim, this study addresses the following research questions: (1) What is the typology and theoretical framework that effectively captures and categorizes the diverse online organizational communication objectives of SA centers as they use Twitter as a strategic tool to achieve their organizational outcomes? (2) How do the sentiments and emotions expressed in Twitter posts by SA centers vary in relation to different categories in the typology of online organizational communication, such as advocacy or public awareness? (3) How can machine learning and content analysis categorize and analyze the social media posts of these organizations, providing insights into their communication strategies?

## Methods

### Overview

**Figure 1:**
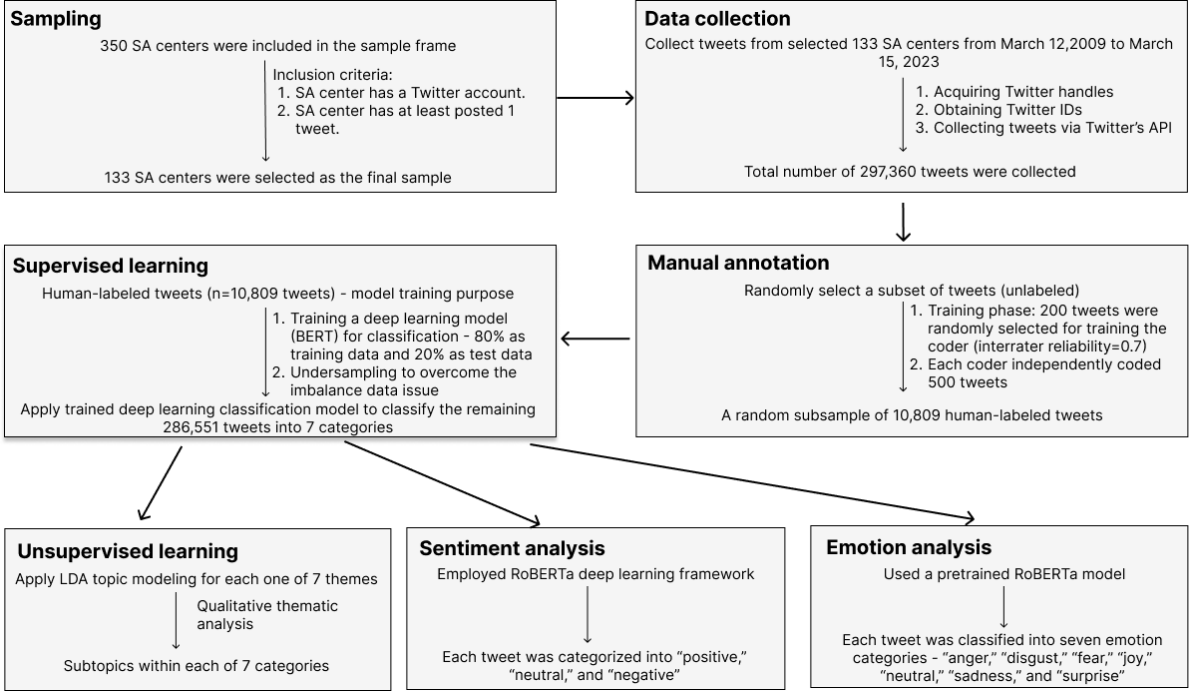
Mixed methods approach. API: application programming interface; LDA: latent Dirichlet allocation; SA: sexual assault.

This study used mixed research methods, including qualitative content analysis, supervised machine learning, unsupervised machine learning, thematic analysis, and sentiment analysis. To classify the full set of tweets, we first manually coded a subset of the full data set (n=10,809 tweets) into 7 emergent categories (Table 1). These human-labeled tweets were used as the training data set to train a supervised machine learning algorithm to classify the remaining tweets. Figure 1 illustrates the mixed methods approach.

**Table 1:** Coding protocol.

Label and classification	Definitions	Tweet samples
1. Community engagement	These tweets involve nonprofits engaging with their community beyond their primary mission. This may include sharing well-wishes, updates on the organization’s activities, quotes or information about books, and other resources that the community might find valuable. Simply providing information about resources is a way to foster engagement.	RT @KidsHelpPhone: “Great article by @OmarMosleh about dealing w/teen grief. Includes quotes from @KidsHelpPhone http://bit.ly/9fqcJn”
2. Organization administration	These tweets pertain to the operation of the organization, such as its operating hours, recruitment of employees and volunteers, organization efforts, information about its activities, and soliciting donations.	“We’ll be closing down the office early in preparation for New Year’s Eve!”
3. Public awareness	These tweets contain information about social, environmental, or economic issues that require attention as well as causes or educational content concerning these issues or causes.	“In North America, 7% of women and 6% of men end up abused by their current or former partners.”
4. Political advocacy	These tweets concern public policy or efforts or initiatives aimed at promoting more far-reaching social change. Based on charitable status, they may not be able to act politically. These tweets may involve the organization’s efforts to draw attention to political issues or comment on government responses referring to what the government is doing to respond to the disaster.	RT @CKNW: “Changes coming to BC family law: Changes are being made to modernize BC’s family law system. Attorney Genera...”
5. Support for others	These tweets pertain to the endorsement of local businesses. Support for others implies backing the initiatives of others that are not directly connected to one’s own mission.	RT @RichmondYouth: “U-Connect Crew is giving back to the community w 3rd annual Winter Holiday Dinner! Volunteer or donate http://ow.ly/NbXP”
6. Partnerships	These tweets relate to the organization’s endeavors to form partnerships and aid other organizations. Partnership involves collaborating with other organizations to further one’s own mission or objective.	@gilmorepark “Happy New Year! Would love to chat with Rev Anna & send over some Outreach & Advocacy brochures for your community suppers.”
7. Appreciation	These tweets concern expressing gratitude toward donors or volunteers for their contributions and dedication toward the organization.	“Best Buy in Richmond has donated a brand new TV to replace the one that broke at Nova Transition House! 3 cheers for @BBYCanada! Thank you!”

### Sampling

To select SA centers in Canada, this study used a purposive sampling approach. Initially, a sampling frame was developed by combining the list of SA centers by province and territory from the Canadian Association of Sexual Assault Centres and the Sexual Assault Centres, Crisis Lines, and Support Services websites. After removing duplicates, the sample frame consisted of 350 SA centers across 10 provinces and 3 territories. The sample frame provided basic information about the centers, including their names, contact information (phone number and email), and website or URL. The inclusion criteria were twofold: (1) the SA center had an active Twitter account and (2) it had posted at least 1 tweet on its account. To verify the eligibility of these centers, the authors manually searched their home page and Twitter pages and conducted thorough Google searches. Ultimately, the Twitter accounts of 133 SA centers were included as the final sample for this study. These centers were from 9 provinces and the Northwest Territories (Prince Edward Island did not have any SA centers that used Twitter).

### Data Collection

To collect tweets from SA centers, the authors followed the pipeline outlined in their papers, including acquiring Twitter handles, obtaining Twitter IDs, and collecting tweets via Twitter’s application programing interface (API) [35-43]. The collected tweets encompassed the period from March 12, 2009, to March 15, 2023. The data set consisted of 297,360 tweets from 133 SA centers in Canada. The data sets are available for use by researchers upon request. First, a total of 91 unique Twitter handles (ie, @name) were obtained from the 133 SA centers in the sample, with 26 duplicate Twitter handles. Second, the 91 Twitter handles were converted into 91 Twitter IDs using 3 websites: TweeterID, CodeOfaNinja, and Comment Picker. Third, Twitter’s premium search API and timeline end points (full-archive end point) were used to collect tweets posted by the sampled SA centers in Canada, starting from as early as 2006 (search tweets, 2019). Data collection concluded on March 15, 2023.

### Manual Annotation

The purpose of manual annotation was to obtain human-labeled tweets categorized into different themes. These labeled tweets would serve as the training data set for classifying the entire corpus using a supervised machine learning approach. The coding protocol was developed based on prior literature on organizational communication research and adapted to suit the objectives of this study. Table 1 included the classification, labels, definitions, and sample tweets.

To ensure consistency, 2 authors (JX and MLS) provided training to the research assistants on the protocol and research goals. During the training phase, a random subset of 200 tweets was selected, and 2 research assistants were assigned to independently code them. This process was repeated 4 times (n=809 tweets) until an acceptable interrater reliability score of 0.7 was achieved for each of the 7 categories. Krippendorff α was used to determine the interrater reliability, which indicated substantial agreement.

Following the training phase, a subset of 10,000 tweets was randomly selected from the collected data. Research assistants were assigned to independently code a subset of 5000 tweets. The manual annotation data set consisted of a random subsample of 10,809 manually labeled tweets categorized into 7 themes from the full data set.

### Construction of Predictive Classification Model

To create an accurate classification model for Twitter data, we used the BERT model [44]. BERT is a widely used natural language processing model that has been pretrained on various English language data sets, making it suitable for fine-tuning tasks such as sentence classification. To evaluate the performance of our model, we randomly selected 80% of the human-labeled tweets as training data, with the remaining 20% used as test data.

Due to the imbalanced distribution of classes in our data set, we used a 2-step strategy to train the machine learning model. First, we fine-tuned the BERT model with all the training data by minimizing the logistic loss [45]. Second, we applied a random undersampling process [46] to retrain the last layer of the BERT model (the classification layer) using this undersampled subset. The undersampling process randomly selected a subset of training data, ensuring an equal number of samples for each class. We chose the undersampling technique as it is less prone to overfitting the data compared to other methods such as oversampling [46].

In addition to using deep learning models in our study, we also used a range of traditional machine learning algorithms as benchmarks for performance comparison. Specifically, we trained models using linear regression, support vector machines with a radial basis function kernel, and support vector machine with a linear kernel. To represent features in these traditional algorithms, we chose the term frequency–inverse document frequency approach to convert our textual data into numerical vectors.

To evaluate the efficacy of these models, we computed the average sensitivity score based on the test data. The sensitivity score for a given class “k” denotes the probability that a sample will be classified by a model as belonging to class “k,” given that the sample truly belongs to that class. We calculated the mean of the sensitivity scores across all 7 classes as our final measurement. Following the training of the BERT model, we used it to classify the unlabeled 286,551 tweets into 7 categories.

### Validation Data

To ensure the robust performance of our model across diverse contexts, we gathered 2 distinct independent data sets from Twitter and Facebook. Independent data set #1 was derived using the same sampling frame in this study. Our aim was to identify organizations active on Facebook but not on Twitter, thereby maintaining uniformity in organization type while varying the social media platform for further model validation. Using Apify software [47], we collected messages from 67 SA organizations and subsequently selected a random sample of 500 messages (n=2520). Independent data set #2 was obtained through a list of human service organizations (approximately 12,000) from the government of Canada’s list of charitable nonprofits (N=85,496). Of the approximately 86,000 charitable nonprofits in Canada, the list of human service organizations was developed through an assessment of the organizations’ website that shows an indication of providing some type of social service programing to a service user group. This frame enabled the identification of organizations with active Twitter accounts, thus ensuring consistency in the chosen social media platform while introducing variation in the type of organization for enhanced model validation. Following the collection of tweets via our API, a random sample of 500 tweets (n=15,696) was selected for data validation. We used the same manual annotation procedure for these 2 data sets to establish manual labels. This allowed us to directly compare the model’s predictions against these manual labels, serving as a method to assess the model’s effectiveness (Multimedia Appendix 1).

### Sentiment Analysis

Sentiment analysis, sometimes referred to as opinion mining, involves the classification and analysis of people’s opinions, sentiments, evaluations, appraisals, attitudes, and emotions concerning various entities, including products, services, organizations, individuals, issues, events, topics, and their associated attributes [48]. Sentiment analysis applied to social media content has been extensively studied, and Twitter has the capability to promptly gauge public sentiments and emotions regarding a given topic [49]. For this analysis, we used RoBERTa, a deep learning framework [50]. We used a pretrained model [51] that was fine-tuned specifically for sentiment analysis of social media data. The model categorized each tweet into 1 of the 3 sentiments: “positive,” “neutral,” or “negative.” We converted a significant amount of textual data into quantitative sentiment scores and calculated the percentage of each sentiment within every category.

### Emotion Analysis

Emotion analysis primarily focuses on capturing nuanced emotions, which contrasts with sentiment analysis, primarily concerned with detecting simple attitudes such as positivity and negativity. Mohammad [52] indicated that machines can infer people’s emotions in a limited way but are useful. We need to hold automatic emotion recognition systems to high standards by incorporating ethical considerations associated with each step of the detection process. In our study, we delve into emotion analysis within 7 categorized groups to investigate and compare potential emotional variations across different categories. For this analysis, we used a pretrained RoBERTa model [53], optimized for emotion analysis of social media data. This model classifies each tweet into 1 of the 7 emotion categories: “anger,” “disgust,” “fear,” “joy,” “neutral,” “sadness,” or “surprise.” We then calculated the percentage of tweets associated with each emotion category for each of our designated categories. Subsequently, we determined the percentage distribution of each emotion within each category. This measurement across various goals and intentions in social media communication provides valuable insights into the perspectives of both the public and SA issues, enhancing our overall understanding of these topics.

### Topic Modeling for Tweets Categorized Into 7 Classes

The objective of this unsupervised machine learning work was to extract latent topics within each theme after categorizing tweets into 7 different categories. To achieve this, we used the latent Dirichlet allocation approach for topic modeling, which allowed us to group tweets into different topics. The initial step in this analysis stage was preprocessing, which enhanced model performance by removing noisy data. We eliminated various elements, such as “mentions,” “emojis,” “hyperlinks,” “RT symbols,” and punctuations, and converted all tweets to lower case. Removing mention symbols eliminated irrelevant terms from the analysis, such as names of organizations and individuals. In addition, we removed stop words such as “the,” “is,” and “and” while retaining nouns, adjectives, and verbs related to events.

Once the data were preprocessed, we implemented latent Dirichlet allocation models using the Gensim library in Python. Our hyperparameter range was set from 1 to 30, and the similarity score served as our evaluation metric. By plotting the similarity score against the number of topics, we identified the turning point on the graph as our optimal hyperparameter. To further analyze the topics within each of the 7 categories, we extracted popular bigrams and reviewed a random sample of tweets. We used qualitative thematic analysis to assign underlying topic meanings to them.

### Topic Evaluation

The team, consisting of domain experts and research assistants, summarized and evaluated the results of the topic modeling. Salient bigrams were used to summarize each topic, and similar topic themes were merged into higher-level categories, as per the machine learning approach described by Zhou et al [54].

### Ethical Considerations

This study used publicly available Twitter data, eliminating the need for ethics approval or consent from organizations. The study data mentioned in this paper underwent processes of anonymization and deidentification. To guarantee full anonymity, all data that could potentially identify individuals or organizations, including users’ metadata and original tweets, have been carefully excluded from the data set.

## Results

### Overview

Our data set included 297,360 tweets and retweets from 133 SA support organizations in Canada. These tweets were posted from March 12, 2009, to March 14, 2023. Multimedia Appendix 2 illustrates a bar plot that summarizes the number of tweets collected for each year.

### Manual Annotation and Class Distribution of Tweets

**Figure 2:**
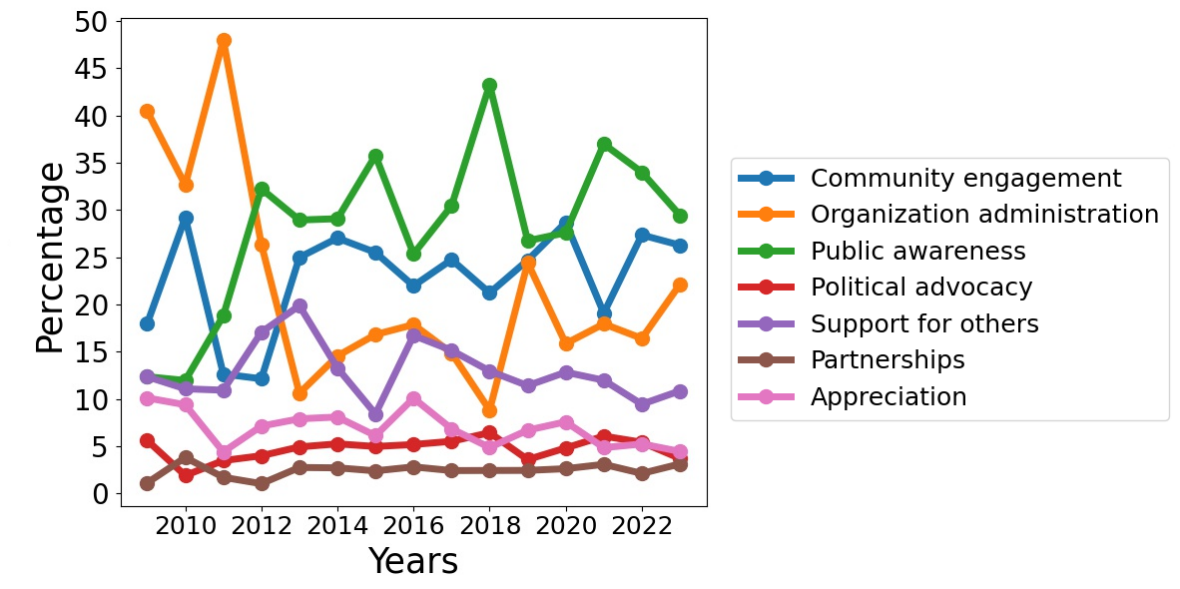
The relationship between the percentage of each category and the corresponding years.

Among the data set consisting of 297,360 tweets and retweets, 10,809 tweets were manually annotated by humans following the coding protocol. As shown in Table 2, organization administration (5652/10,809, 52.29%) was the most frequent type of post, followed by community engagement (2322/10,809, 21.38%) and public awareness (1522/10,809, 14.08%). The smallest segment of tweets belonged to political advocacy (129/10,809, 1.19%) and partnerships (162/10,809, 1.5%). Multimedia Appendix 3 presents the histogram of class distributions of the human-labeled tweets. It is worth noting that the distribution of labels was imbalanced, with a smaller proportion of tweets (<5%) falling into the categories of political advocacy, support for others, and partnerships. Figure 2 presents a plot showing the percentage of each category plotted against the corresponding years.

**Table 2:** Comparison of human-annotated and machine learning classified tweets (N=297,360).

	Human annotation (n=10,809), n (%)	Supervised machine learning model (n=286,551), n (%)
Community engagement	2311 (21.38)	67,285 (23.5)
Organization administration	5652 (52.29)	50,620 (17.7)
Public awareness	1522 (14.08)	89,295 (31.2)
Political advocacy	129 (1.19)	14,504 (5.1)
Support for others	288 (2.66)	38,083 (13.3)
Partnerships	162 (1.5)	7,144 (2.5)
Appreciation	745 (6.89)	19,620 (6.8)

### Performance of the Supervised Machine Learning Model

We evaluated the performance of the supervised machine learning model using the test set (20% of the human-labeled tweets). The accuracy of machine learning classification achieved by our trained model was 63.4%, which was higher than that of the human coders who labeled the training data set. This indicates an improvement in the BERT model’s ability to accurately predict classifications compared to those made by human coders.

We analyzed and presented the confusion matrices in Tables 3 and 4. Table 3 displays the confusion matrix for the initial model without undersampling, whereas Table 4 illustrates the confusion matrix with undersampling applied. These matrices provide insights into the percentage of samples with actual labels that were correctly classified into the predicted class by the model. The sensitivity scores for each class are represented on the diagonal of the matrices, and the average sensitivity score across the 7 classes serves as an indicator of the overall performance of the model. As observed in the tables, the average sensitivity scores improved from 48.3% to 53.1%. As demonstrated in tables, BERT’s performance surpassed that of traditional machine learning methods.

**Table 3:** Confusion matrix for the initial model without undersampling.

			Predicted classes (%)											
			Community engagement		Organization administration	Public awareness		Political advocacy		Support for others		Partnerships		Appreciation
**Actual classes**														
	Community engagement	59.3		21.8		12.7	0.4		2.2		0.3		3.3	
	Organization administration	6.6		87.6		2.7	0.4		0.8		0.3		1.7	
	Public awareness	20.1		16.6		60.7	0.7		1.1		0.0		0.9	
	Political advocacy	22.6		24.2		38.7	12.9		1.6		0.0		0.0	
	Support for others	20.5		44.3		14.8	0.0		11.5		0.8		8.2	
	Partnerships	5.0		45.0		5.0	0.0		0.0		37.5		7.5	
	Appreciation	6.3		16.1		4.6	0.0		2.9		1.1		69.0	

**Table 4:** Confusion matrix for initial model with undersampling.

			Predicted classes (%)											
			Community engagement		Organization administration	Public awareness		Political advocacy		Support for others		Partnerships		Appreciation
**Actual classes**														
	Community engagement	57.0		11.4		12.7	3.6		11.4		1.6		2.3	
	Organization administration	8.2		75.8		3.3	1.3		7.2		2.7		1.4	
	Public awareness	17.4		9.1		58.9	5.1		6.8		1.5		1.1	
	Political advocacy	9.7		14.5		32.3	40.3		3.2		0.0		0.0	
	Support for others	20.5		25.4		13.1	1.6		30.3		5.7		3.3	
	Partnerships	2.5		20.0		0.0	0.0		10.0		55.0		12.5	
	Appreciation	5.7		9.8		4.0	0.6		13.2		5.2		61.5	

### Results of Predictive Classification of Unlabeled Data Using Machine Learning

The remaining 286,551 tweets were classified into 7 classes using the supervised machine learning algorithms. The classification results are presented in Table 2. The supervised machine learning model produced classifications that were similar to those of the human-annotated tweets in terms of the percentage of each category. The most frequent tweet class was public awareness (89,295/286,551, 31.2%), followed by community engagement (67,285/286,551, 23.5%) and organization administration (50,620/286,551, 23.5%). The 2 smallest categories of posts were partnerships (7144/286,551, 2.5%) and political advocacy (19,620/286,551, 6.8%).

### Top Unigrams and Bigrams in the Tweets

We conducted an analysis to identify the most commonly used words and phrases in the tweets from the SA support organizations. To do this, we removed the stop words and generated a list of the top 30 most frequently occurring unigrams and bigrams, as presented in Multimedia Appendices 4 and 5. We observed that >220,000 tweets included a URL link, which directed users to news or events related to SA. In addition, approximately 100,000 tweets were retweets, with “rt” in the messages. The terms “women,” “support,” “sexual assault,” “sexual violence,” and “crisis line” were among the most commonly used terms in these tweets.

### Sentiment and Emotion Analysis Results

We conducted sentiment and emotion analysis, and the summarized results can be found in Tables 5 and 6. The findings revealed that the neutral sentiment category surpassed both the negative and positive sentiment categories across all 7 classes.

Regarding the emotion analysis, most tweets from organizations were associated with the emotion of “fear.” In contrast, tweets discussing topics related to class 6 (partnerships) and class 7 (appreciation) exhibited the emotion of “joy.” Here are a few examples of tweets reflecting fear:

Every minute of every day, a Canadian woman or child is being sexually assaulted. #VAW

Salau’s story is so symbolic of how universally disregarded, disrespected, and unprotected Black women are, even in our most vulnerable moments. #EndVAW #JusticeForToyin.

Here is a tweet reflecting joy (appreciation):

We’ve seen that charity brings together amazing people to create great change and make meaningful impact in the lives of the people in their community. THANK YOU to the incredible supporters who make our work possible...

These examples illustrate the emotional tone associated with different tweet categories, with fear being prevalent among organizational tweets and joy being linked to discussions on partnerships and appreciation.

**Table 5:** The sentiments for each category.

	Negative (%)	Neutral (%)	Positive (%)
Community engagement	6.7	72.2	21.1
Organization administration	0.5	91.8	7.6
Public awareness	18.4	80.3	1.4
Political advocacy	14.7	82.0	3.3
Support for others	1.3	69.4	29.4
Partnerships	0.3	73.9	25.8
Appreciation	0.6	25.8	73.6

**Table 6:** The emotions for each category.

	Anger (%)	Disgust (%)	Fear (%)	Joy (%)	Neutral (%)	Sadness (%)	Surprise (%)
Community engagement	4.5	1.1	52.3	12.6	24.5	3.1	1.9
Organization administration	1.5	0.1	59.3	10.9	24.8	2.1	1.3
Public awareness	8.7	3.2	72.2	1.3	10.1	3.9	0.6
Political advocacy	8.2	2.4	65.3	3.4	16.3	3.5	0.9
Support for others	3.8	0.1	59.3	21.4	11.1	1.7	2.5
Partnerships	2.1	0.1	53.4	27.6	13.6	0.7	2.4
Appreciation	3.8	0.0	34.6	54.8	4.3	0.9	1.5

### Topic Modeling Results

The coded data set yielded distinct topics within each of the classes or categories. The identified topics, bigrams, and representative tweet examples are presented in Table 7. These themes provide insights into the prevalent topics and discussions within the data set, showcasing different aspects of the discourse surrounding SA and related issues.

**Table 7:** Sexual assault organizations tweets by topics.

Topics within the category		Bigrams (top 3-5)	Example tweet
**1. Community engagement**			
	Experience and awareness of abuse	experience abuse, wear purple, everyday believe, know experience, red flag, woman know	RT @SHORECentreWR: “Don’t forget to wear your purple today!”
	Support and information	post photo, need help, human right, safe way, information support, self-care, free violence	“Nova Scotians are grieving. Mandalas are a symbol of healing with roots in ancient cultural traditions. We invite you to create a mandala, photograph it, and send the photo to...will post the images to Instagram...”
	Social media engagement	social media, friend family, find way, medium account, check pic, keep connected	“Great convo going on among Centre residents: social media and sharing too much information. Program like this supports personal growth”
**2. Organization administration**			
	Sexual assault support services and helplines	support line, service available, online phone, crisis line, toll free	“...you want help figuring out a safe warm place to sleep. That phone line is staffed 24/7. We know this is a bandaid and not a permanent solution, but for now we’re focused on safety from this dangerous cold...”
	Support groups and emotional support	support group, need help, join team, ticket sale, dropin support	“In response to requests from folks using the space, we decided to make Wednesdays a drop-in for women, trans*, and non-binary persons only. Monday mornings will continue to be open to all genders”
**3. Public awareness**			
	Gender-based violence and advocacy	Genderbased, indigenous woman, woman kill, remember woman	RT @kevinkindred: “Given today’s news that there is no evidence of unmarked graves at the Shubenacadie residential school in NS, let’s take a moment to remember the names of the sixteen indigenous children known to have died at the school”
	Sexual assault awareness and support	sexual assault, sexual abuse, experience sexual, rape culture	—^a^
	Violence against women and rights advocacy	violence woman, support survivors, end violence, human right	@JulieSLalonde @ArielTroster “There are women right now in violent relationships who feel unsafe at home and are not leaving because there’s no place to go.”
**4. Political advocacy**			
	Advocacy for survivors of sexual assault and justice	end violence, fund sexual, sign petition, criminal justice, assault law, federal government	RT @Pam_Palmater: “Join Dr. @cblackst &; I on YouTube LIVE Friday, Dec. 2nd at 2:00pm ET to get an update on federal gov/mt &; @AFN_Updates decision to file for judicial review of Can. Human Rights Tribunal decision to protect compensation order for First Nations kids.”
	Legal aid for survivors of sexual assault	justice system, pour femme, femme service, national inquiry, coalition rape, legal aid	“We have continued to mask and offer testing all along. It’s not a political statement, it’s simply because we care about public health, about each other and everyone in our community. \np.s. it’s really not that hard to do.”
	Indigenous rights and reconciliation	indigenous people, action reconciliation, canadian history, justice system, provincial government	RT @JarvisGoogoo: “At @SchulichLaw in March 2005 re Indigenous law, Candy Palmater said that if White women faced violence at the same rate as Aboriginal women did in this country, Canada would declare a national state of emergency”
	Sexual violence and action for change	call action, reconciliation justice, truth stand, action reconciliation	RT @CSaulnierHfx: “I feel that there’s a growing collective spirit that is calling for political change, political action on this issue.” @FranklyLess #EndPoverty #nspoli @hfxexaminer
**5. Support for others**			
	Support and advocacy for survivors of sexual assault	mental health, community health, make difference, public education, training forum	RT @SVPWR: “It’s #BellLetsTalk today, and we are so grateful for the local organizations doing so much to support mental health in #WatReg:...”
	Campaigns and events to raise awareness about sexual assault	purple today, purple support, executive director, social medium, campaign launch, spread word	RT @welcomehousing: “Tomorrow is @worldpancreatic day &; we will be wearing purple for our late coordinator, Bryon Anderson #WPD15...”
**6. Partnerships**			
	Support and fundraising for survivors of sexual violence	support survivors, raise funds, proceed donate, annual fundraiser, community partners, local business	“Special people deserve a special shout out. Tarek, Karen and their crew are working today to prepare and donate 120 hot meals. This is such a beautiful gift from Tarek’s. We’re taking the meals to individuals and families we support in hotels because #Halifax shelters are full.”
	Campaigns and support for ending violence against women	support woman, support survivor, support service, raise money, raise awareness, supporting campaigns	“We are excited to be co-hosting an IWD Event, March 8th, 6:30 pm Atlantic with The Local Council of Women Halifax. Join us for ‘Addressing Women\’s Housing Needs in Halifax: Exposing the intersections of invisibility.’ Register for the event here:”
	Programs and efforts to support women and children	download app, effort download, app gather	“Western researchers will be leading the way in developing the first free, Canada-wide app to help survivors of domestic violence find a path to safety and health. #innovation #stopdomesticviolence”
	Community engagement and support	Join today, raise money, join tomorrow, silent auction	“We have many many community partners and our amazing show-stopping student Sam has made gifts to say thanks. Deliveries en route.”
**7. Appreciation**			
	Gratitude and appreciation for support	thank support, thank help, spread the word, silent auction, thank come, big thank	“@awarham thank you, a lot of people contributed their wisdom, knowledge and experience. and we've had fantastic professional, moral and financial support ❤️🌻 without all that, we wouldn’t be here.”
	Thanking supporters and donors	generous donation, thank donate	“To the anonymous donor, thank you for this #RAK Comfort food for shelter residents on the heels of heavy news #NovaScotia #COVID19NS Also a great way to support local #shoplocal”
	Gratitude for engagement and participation	Big thank, thank share, huge thank, walk mile, support survivors, mile shoe	“The walk has officially ended and we thank everyone who volunteered and walked the walk!!! #rrwalk2013”

^a^Not available.

### Community Engagement

Approximately 20% of the tweets in the data set contained themes related to community engagement, generating 3 topics: “experience and awareness of abuse,” “support and information,” and “social media engagement.” Topic 1 focuses on discussions related to experiences of abuse and raising awareness about it. Topic 2 revolves around providing support and information and promoting human rights in relation to SA. Topic 3 highlights engagement on social media platforms, connecting with friends and family, and finding ways to stay informed and connected.

### Organization Administration

In the organization administration class, “sexual assault support services,” “helplines,” and “support groups and emotional support” were the salient topics. The first topic revolves around providing support for survivors of SA, and the tweets likely contain information about available services and crisis lines; promote helpline numbers; and emphasize the availability of support services. Topic 2 centers on support groups and emotional support for individuals impacted by sexual violence. The tweets may discuss the importance of support networks, encourage individuals to join support groups, and highlight the emotional support available.

### Public Awareness

In the public awareness class, we identified 3 topics. Topic 1 focuses on discussions related to gender-based violence, and the tweets likely highlight the need to raise awareness, advocate for survivors, and address issues surrounding gender-based violence. Topic 2 centers on SA awareness, support for survivors, and efforts to combat sexual violence. The tweets may highlight initiatives such as awareness months, support services, survivor empowerment, and the importance of ending sexual violence. Topic 3 focuses on violence against women, advocating for women’s rights, and addressing issues such as domestic violence and intimate partner violence. The tweets may discuss the importance of human rights, raise awareness about violence against women, and emphasize the need for support services.

### Political Advocacy

This theme delves into the criminal justice system’s response to SA cases and advocates for changes and reforms. It discusses specific initiatives, such as signing petitions and calling for justice for communities considered marginalized. It also mentions the importance of advocacy and policy work for Francophone women. Topic 1 revolves around discussions related to SA, advocating for survivors, and seeking justice. The tweets may address issues such as domestic violence, support for survivors, legal cases, and the need for systemic change in addressing SA and violence against women. Topic 2 focuses on support services and resources available for survivors of SA, including centers providing assistance and legal aid. The tweets may mention crisis centers, justice systems, confidential services, and the importance of providing support and resources to survivors. Topic 3 highlights discussions surrounding Indigenous rights, reconciliation, and addressing SA and violence within Indigenous communities. The tweets may emphasize actions for truth and reconciliation, support for Indigenous women, and the need for systemic change to combat violence and promote gender equality. Topic 4 addresses sexual violence in general, including domestic violence and violence against women. The tweets may focus on the need for action, standing against violence, justice systems, systemic change, and the role of government in addressing sexual violence.

### Support for Others

This theme included 2 topics, highlighting the importance of community involvement, support, and collective efforts to combat violence and abuse. Topic 1 emphasizes “support and advocacy for survivors of sexual assault,” and the tweets may mention initiatives, organizations, and individuals working to support and address their mental health needs, highlighting the importance of community efforts in making a positive difference. Topic 2 focuses on the “campaigns and events to raise awareness about sexual assault,” and the discussion within this topic often involves various campaigns and events, with tweets possibly mentioning actions such as wearing purple to demonstrate support as well as sharing information and promoting community campaigns.

### Partnerships

This theme centers on the importance of partnerships, fundraising efforts, and collective efforts to address and prevent violence and abuse. We identified 4 topics within the theme. Topic 1 revolves around providing support and raising funds for survivors of sexual violence. The tweets may mention community partners, local businesses, and fundraising efforts aimed at supporting survivors. Topic 2 focuses on campaigns and initiatives to end violence against women. The tweets may mention supporting campaigns, raising funds, and providing support services for survivors of SA. The topic highlights the importance of community engagement and collective action in addressing violence against women. Topic 3 emphasizes programs and efforts aimed at supporting women and children who have experienced SA. The tweets may mention fundraising events, supporting local services, helplines, and providing assistance to survivors. Topic 4 revolves around community engagement and support related to sexual violence. The tweets may mention joining teams, spreading the word, and supporting survivors through initiatives such as silent auctions. The topic highlights the importance of community participation and collaboration in addressing sexual violence.

### Appreciation

The least common theme revealed 3 topics, including “gratitude and appreciation for support,” “thanking supporters and donors,” and “gratitude for engagement and participation.” This theme revolves around expressing gratitude and appreciation for support, donations, and contributions. Topic 1 revolves around expressing gratitude and appreciation for the support received. The tweets thank individuals, organizations, and community members, and the topic emphasizes the importance of acknowledging and recognizing the contributions of supporters. Topic 2 focuses on thanking supporters and donors for their contributions. The tweets express gratitude toward individuals and organizations for their generous donations and ongoing support. The topic highlights the significance of recognizing and thanking those who have contributed to the cause. Topic 3 focuses on expressing gratitude for engagement and participation. The tweets thank individuals for sharing information, participating in events such as walks or auctions, and making a difference. The topic emphasizes the importance of community involvement and active participation.

## Discussion

### Principal Findings

This study presents a comprehensive classification of Twitter messages that elucidate the reasons for social media use among SA support centers in Canada. Leveraging a data set of 297,360 tweets from 133 SA support organizations, the application of supervised machine learning enabled us to automatically predict content analytical themes within the Twitter corpus. First, we identified the emerging classifications of Twitter’s use by human service nonprofits. The results indicated that Twitter is used by SA centers across Canada for various purposes. The identified classifications include (1) community engagement, (2) organization administration, (3) public awareness, (4) political advocacy, (5) support for others, (6) partnerships, and (7) appreciation. These categories reflect the multifaceted nature of human service nonprofits’ communication strategies and their engagement with stakeholders. Second, the findings of this study contribute to the existing literature by expanding the understanding of social media use by human service nonprofits beyond the traditional focus on advocacy-related purposes. Although advocacy remains an important aspect, this research reveals that these organizations use social media to achieve a diverse range of objectives, such as raising public awareness, community engagement, and organization administration. Third, the sentiment and emotion analysis of tweets shed light on the emotional tone of different tweet categories. The prevalence of “fear” among organizational tweets underscores the gravity of the issues addressed by human service nonprofits. In contrast, the emotion of “joy” associated with the partnership and appreciation categories highlights the positive impact of community involvement and support. Fourth, the application of machine learning in this study has proven to be valuable in predicting content analytical themes in a large Twitter corpus. The predictive classification model outperformed human coders in terms of accuracy, indicating the potential of machine learning algorithms in analyzing social media data and gaining deeper insights into nonprofits communication strategies.

### Typology and Theoretical Framework of Online Organizational Communication Objectives

A key discovery from our study pertains to the distribution of tweet categories. The analysis revealed that the most frequent type of posts falls under the “public awareness” category. Approximately one-third of the collected tweets were classified within this category, signifying that SA support organizations predominantly use social media platforms for advocating against issues related to intimate partner violence and sexual violence. These findings align with prior literature, highlighting how social media allows organizations to disseminate content aimed at increasing awareness while incurring minimal costs [8]. Our topic modeling results uncovered 3 salient themes within the public awareness category, which encompass tweets that emphasize the need to raise awareness, advocate for addressing gender-based violence issues, and support survivors of sexual violence and women’s rights. This emphasis on awareness-raising activities reflects the pivotal role social media plays in creating awareness for organizations and fostering interactions with donors and volunteers [55,56].

Community engagement emerges as the second most prominent reason for social media use by SA organizations in Canada, constituting approximately one-fourth of all collected tweets. This category generated 3 distinct topics through classifications, where community engagement entails nonprofits engaging with their community beyond their primary mission. These tweets include sharing well-wishes, updates on organization activities, quotes, and information about resources that the community may find valuable. Although some tweets do touch on providing information beyond their primary mission, the main focus remains on community engagement concerning sexual violence support. The nuanced examination of tweet contents provided by our topic modeling analysis sheds light on the multifaceted nature of community engagement efforts by SA support organizations. It is evident that these organizations recognize the significance of engaging with their communities regarding sexual violence and related matters through social media. Consistent with existing literature, our findings align with the view that social media offers an avenue for powerful participation and community engagement [57]. It also emphasizes the potential role of social media as a mechanism to raise awareness and inform the community of various initiatives and projects [58]. Although community engagement through social media remains an opportunity for SA support organizations to connect with their communities and market their services, further research should delve into the effectiveness of engagement across all aspects of design, delivery, and evaluation, particularly with regard to specific objectives such as supporting and combating sexual violence.

### Sentiments and Emotions in Different Categories in the Typology

Sentiment analysis is used to examine the evaluative perspectives expressed within the text, with its importance stemming from its ability to comprehend the shifting dynamics, potential interventions, and predictive insights into public sentiment regarding trending events. It serves as a valuable tool for offering decision-making support to relevant authorities in the realms of public sentiment monitoring, intervention, and governance. Our sentiment and emotion analysis yielded valuable insights into the emotional tone of the tweets related to each identified online organizational communication objective. Most tweets exhibited a neutral sentiment, surpassing both the negative and positive categories across all 7 classes. This prevalence of neutrality could be attributed to the sensitive nature of the topic, as SA is a deeply distressing issue. However, it is worth noting that tweets related to partnerships and appreciation displayed a greater presence of joy as the dominant emotion, indicating positive sentiments associated with community engagement, support, and expressions of gratitude.

### Machine Learning Classification

The evaluation of the machine learning model’s performance on the test set showcased promising results. The model demonstrated improvement in average sensitivity scores, from 48.3% to 53.1%, indicating its ability to accurately classify tweets into their respective categories and provide reliable predictions for content analytical themes. Nonetheless, it is essential to acknowledge that there is still room for improvement in the model’s performance, particularly in accurately classifying tweets related to partnerships and political advocacy, which constituted smaller segments of the data set.

### Implications

Our research carries significant implications for both practitioners and policy makers in the field of SA support services. The typology we have developed represents a substantial advancement in research within this domain and provides a comprehensive framework for understanding how these organizations can effectively use Twitter to disseminate information and engage with the public at the message level. This typology empowers human service nonprofits to align their social media strategies with specific organizational goals. By understanding the different categories and topics of Twitter communication, these organizations can tailor their content and engagement strategies to maximize the impact and relevance of their online presence.

The study emphasizes the importance of social media as a potent communication tool for engaging with communities, raising public awareness, and providing essential information and support. Through an understanding of the diverse communication themes and strategies used by SA support organizations, practitioners can optimize their social media use to effectively reach and connect with their target audience.

On the basis of our research findings, we recommend that human service nonprofits invest in social media education and training for their personnel to enhance their understanding of how to use social media effectively. By building a strategic approach to social media use that aligns with organizational objectives, these nonprofits can maximize their impact and outreach, ultimately furthering their mission to support and advocate for survivors of SA.

### Limitations

The study has certain limitations that need to be acknowledged. The predictive performance of the model is influenced by factors such as human annotation, interrater agreement, and the training data set. The trained model attempts to mimic the classification by the human coders, whose understanding of the tweet content and familiarity with the background and theoretical framework is critical to the study. In this regard, the study underwent 4 rounds of training to attain a satisfactory interrater reliability score. However, future studies could incorporate more human coders to enhance the accuracy of the results. Furthermore, our analysis focused solely on Twitter data from SA support organizations in Canada. This geographic and platform limitation may restrict the generalizability of our findings to other countries and social media platforms. Future research should consider expanding the scope to include a more diverse range of organizations and platforms. Finally, although our machine learning model demonstrated promising performance, there is still room for improvement. The accuracy of the model in classifying tweets related to partnerships and political advocacy was relatively lower compared to other categories. Further refinement and fine-tuning of the model could enhance its accuracy and reliability.

### Conclusions

In conclusion, our study offers valuable insights into the application of machine learning to understand the message-level communication purposes of SA support organizations on Twitter in Canada. By combining social science and computer science, we effectively analyzed a large data set and identified content analytical themes, sentiments, emotions, and topics within tweets. These findings enrich our understanding of how SA support organizations use social media for community engagement, public awareness, and organizational administration purposes. The implications extend to practitioners, policy makers, and organizational personnel, emphasizing the significance of education and training to maximize the benefits of social media in achieving organizational goals within the realm of SA support services.

## Appendix

Multimedia Appendix 1 Confusion matrix for the independent data sets.

Multimedia Appendix 2 The number of tweets collected each year.

Multimedia Appendix 3 The histograms of the categories in the human-labeled data: (1) community engagement, (2) organization administration, (3) public awareness, (4) political advocacy, (5) support for others, (6) partnerships, and (7) appreciation.

Multimedia Appendix 4 Top unigrams in the tweets.

Multimedia Appendix 5 Top bigrams in the tweets.

## Abbreviations

API: application programing interface
SA: sexual assault

## Data Availability

The data sets generated during and analyzed during this study are available from the corresponding author on reasonable request.

## References

[1] Enjolras B (2022). Determinants of voluntary organizations’ attention on Facebook: the case of Norwegian voluntary organizations. Nonprofit Volunt Sect Q.

[2] Lai CH, Fu JS (2020). Humanitarian relief and development organizations’ stakeholder targeting communication on social media and beyond. Voluntas.

[3] Xu W, Saxton GD (2018). Does stakeholder engagement pay off on social media? A social capital perspective. Nonprofit Volunt Sect Q.

[4] Bhati A, McDonnell D (2019). Success in an online giving day: the role of social media in fundraising. Nonprofit Volunt Sect Q.

[5] Priante A, Ehrenhard ML, van den Broek T, Need A, Hiemstra D (2021). “Mo” together or alone? Investigating the role of fundraisers’ networks in online peer-to-peer fundraising. Nonprofit Volunt Sect Q.

[6] Zhou H, Ye S (2018). Legitimacy, worthiness, and social network: an empirical study of the key factors influencing crowdfunding outcomes for nonprofit projects. Voluntas.

[7] Zhou H, Ye S (2019). Fundraising in the digital era: legitimacy, social network, and political ties matter in China. Voluntas.

[8] Bilgin Y, Kethüda Ö (2022). Charity social media marketing and its influence on charity brand image, brand trust, and donation intention. Voluntas.

[9] Livermore M, Verbovaya O (2016). Doing collaboration: how organizations use Facebook to foster collaboration. Hum Serv Org Manage Leadership Gov.

[10] Campbell DA, Lambright KT (2019). Are you out there? Internet presence of nonprofit human service organizations. Nonprofit Volunt Sect Q.

[11] Guo C, Saxton GD (2013). Tweeting social change: how social media are changing nonprofit advocacy. Nonprofit Volunt Sect Q.

[12] Halpin DR, Fraussen B, Ackland R (2020). Which audiences engage with advocacy groups on Twitter? Explaining the online engagement of elite, peer, and mass audiences with advocacy groups. Nonprofit Volunt Sect Q.

[13] Sitter KC, Curnew AH (2016). The application of social media in social work community practice. Soc Work Educ.

[14] Shier ML, Handy F (2014). From advocacy to social innovation: a typology of social change efforts by nonprofits. Voluntas.

[15] Cooner TS, Beddoe L, Ferguson H, Joy E (2019). The use of Facebook in social work practice with children and families: exploring complexity in an emerging practice. J Technol Hum Serv.

[16] O'Sullivan L, Hughes Z (2019). Incorporating Facebook into nonprofit supports for family caregivers: reflections on its value and relevance. J Technol Hum Serv.

[17] Tian F, Labban A, Shearer R, Gai Q (2019). The impact of social media activity on nonprofit donations in China. Voluntas.

[18] Zorn TE, Grant S, Henderson A (2012). Strengthening resource mobilization chains: developing the social media competencies of community and voluntary organizations in New Zealand. Voluntas.

[19] Comfort SE, Hester JB (2019). Three dimensions of social media messaging success by environmental NGOs. Environ Commun.

[20] Lam WF, Nie L (2019). Online or offline? Nonprofits’ choice and use of social media in Hong Kong. Voluntas.

[21] Svensson PG, Mahoney TQ, Hambrick ME (2014). Twitter as a communication tool for nonprofits: a study of sport-for-development organizations. Nonprofit Volunt Sect Q.

[22] Guo C, Saxton GD (2017). Speaking and being heard: how nonprofit advocacy organizations gain attention on social media. Nonprofit Volunt Sect Q.

[23] Chan C (2016). A scoping review of social media use in social work practice. J Evid Inf Soc Work.

[24] Goldkind L (2015). Social media and social service: are nonprofits plugged in to the digital age?. Hum Serv Org Manage Leadership Gov.

[25] Young JA (2016). Facebook, Twitter, and blogs: the adoption and utilization of social media in nonprofit human service organizations. Hum Serv Org Manage Leadership Gov.

[26] Ihm J, Kim E (2021). When nonprofit organizations meet information and communication technologies: how organizational culture influences the use of traditional, digital, and sharing media. Voluntas.

[27] Seo H, Vu HT (2020). Transnational nonprofits’ social media use: a survey of communications professionals and an analysis of organizational characteristics. Nonprofit Volunt Sect Q.

[28] Huang S (2022). NGO as sympathy vendor or public advocate? A case study of NGOs’ participation in internet fundraising campaigns in China. Voluntas.

[29] Meijer A (2012). Co-production in an information age: individual and community engagement supported by new media. Voluntas.

[30] Wu VC (2022). Exploring donor influence and public engagement: computational and thematic analyses of social media messages. Voluntas.

[31] Byrne J, Kirwan G, Mc Guckin C (2019). Social media surveillance in social work: practice realities and ethical implications. J Technol Hum Serv.

[32] Beaumont E, Chester P, Rideout H (2017). Navigating ethical challenges in social media: social work student and practitioner perspectives. Aus Soc Work.

[33] Voshel EM, Wesala A (2015). Social media and social work ethics: determining best practices in an ambiguous reality. J Soc Work Values Ethics.

[34] Stanfield D (2020). Social media and social work education curriculum in Aotearoa New Zealand: an integrated framework. Adv Soc Work Welfare Educ.

[35] Xue J, Chen J, Hu R, Chen C, Zheng C, Su Y, Zhu T (2020). Twitter discussions and emotions about the COVID-19 pandemic: machine learning approach. J Med Internet Res.

[36] Xue J, Chen J, Chen C, Zheng C, Li S, Zhu T (2020). Public discourse and sentiment during the COVID 19 pandemic: using Latent Dirichlet Allocation for topic modeling on Twitter. PLoS One.

[37] Xue J, Chen J, Chen C, Hu R, Zhu T (2020). The hidden pandemic of family violence during COVID-19: unsupervised learning of tweets. J Med Internet Res.

[38] Su Y, Xue J, Liu X, Wu P, Chen J, Chen C, Liu T, Gong W, Zhu T (2020). Examining the impact of COVID-19 lockdown in Wuhan and Lombardy: a psycholinguistic analysis on Weibo and Twitter. Int J Environ Res Public Health.

[39] Xiang X, Lu X, Halavanau A, Xue J, Sun Y, Lai PH, Wu Z (2021). Modern senicide in the face of a pandemic: an examination of public discourse and sentiment about older adults and COVID-19 using machine learning. J Gerontol B Psychol Sci Soc Sci.

[40] Xue J, Chen J, Gelles R (2019). Using data mining techniques to examine domestic violence topics on Twitter. Violence Gender.

[41] Xue J, Macropol K, Jia Y, Zhu T, Gelles RJ (2019). Harnessing big data for social justice: an exploration of violence against women‐related conversations on Twitter. Hum Behav Emerg Tech.

[42] Zheng C, Xue J, Sun Y, Zhu T (2021). Public opinions and concerns regarding the Canadian Prime Minister's daily COVID-19 briefing: longitudinal study of YouTube comments using machine learning techniques. J Med Internet Res.

[43] Xue J (2018). Agenda-setting for intimate partner violence: exploring the role of social media United States-based Twitter. University of Pennsylvania.

[44] Devlin J, Chang MW, Lee K, Toutanova K BERT: pre-training of deep bidirectional transformers for language understanding. arXiv.

[45] Brownlee J (2019). Probability for Machine Learning: Discover How To Harness Uncertainty With Python.

[46] Japkowicz N, Stephen S (2022). The class imbalance problem: a systematic study. Intell Data Anal.

[47] Build reliable web scrapers. Fast. Apify.

[48] Liu B (2012). Sentiment Analysis and Opinion Mining.

[49] Eysenbach G (2011). Infodemiology and infoveillance tracking online health information and cyberbehavior for public health. Am J Prev Med.

[50] Liu Y, Ott M, Goyal N, Du J, Joshi M, Chen D, Levy O, Lewis M, Zettlemoyer L, Stoyanov V RoBERTa: a robustly optimized BERT pretraining approach. arXiv.

[51] Hartmann J, Heitmann M, Schamp C, Netzer O (2021). The power of brand selfies. J Mark Res.

[52] Mohammad SM (2022). Ethics sheet for automatic emotion recognition and sentiment analysis. Comput Linguist.

[53] Emotion English DistilRoBERTa-base. Hugging Face.

[54] Zhou S, Zhao Y, Bian J, Haynos AF, Zhang R (2020). Exploring eating disorder topics on Twitter: machine learning approach. JMIR Med Inform.

[55] Cahalane C (2013). Charities should be proactive and approach local authorities. The Guardian.

[56] Quinton S, Fennemore P (2012). Missing a strategic marketing trick? The use of online social networks by UK charities. J Philanthr Mark.

[57] Ross C, Warwick C, Terras M, Nyhan J (2012). Social media for digital humanities and community engagement. Digital Humanities in Practice.

[58] Steinmetz C, Rahmat H, Marshall N, Bishop K, Thompson S, Park M, Corkery L, Tietz C (2020). Liking, tweeting and posting: an analysis of community engagement through social media platforms. Urban Policy Res.

