# Supplementary material for: A Typology of Social Media Use by Human Service Nonprofits: Mixed Methods Study
Source: J Med Internet Res. 2024 May 8;26:e51698. doi: 10.2196/51698 (PMC11112479; doi:10.2196/51698)
Supplement: Multimedia Appendix 1 [file jmir_v26i1e51698_app1.docx]

**Appendix 1**

**Confusion Matrix for the independent Facebook dataset (n=500).**

|  | Pred = 1 | Pred = 2 | Pred = 3 | Pred = 4 | Pred = 5 | Pred = 6 | Pred = 7 |
| --- | --- | --- | --- | --- | --- | --- | --- |
| Label = 1 | 63.2% | 8.8% | 14.0% | 5.3% | 7.0% | 1.8% | 0.0% |
| Label = 2 | 3.7% | 86.4% | 2.5% | 1.9% | 2.5% | 1.9% | 1.2% |
| Label = 3 | 9.4% | 2.1% | 81.2% | 1.0% | 4.2% | 0.0% | 2.1% |
| Label = 4 | 3.5% | 22.8% | 52.6% | 15.8% | 5.3% | 0.0% | 0.0% |
| Label = 5 | 20.4% | 34.7% | 4.1% | 4.1% | 26.5% | 2.0% | 8.2% |
| Label = 6 | 16.7% | 8.3% | 0.0% | 0.0% | 0.0% | 75.0% | 0.0% |
| Label = 7 | 4.5% | 0.0% | 0.0% | 0.0% | 1.5% | 0.0% | 94.0% |

**Confusion Matrix for independent twitter dataset (n=500).**

|  | Pred = 1 | Pred = 2 | Pred = 3 | Pred = 4 | Pred = 5 | Pred = 6 | Pred = 7 |
| --- | --- | --- | --- | --- | --- | --- | --- |
| Label = 1 | 46.9% | 20.4% | 8.2% | 6.1% | 12.2% | 2.0% | 4.1% |
| Label = 2 | 2.8% | 87.0% | 1.1% | 0.6% | 5.1% | 2.8% | 0.6% |
| Label = 3 | 15.0% | 2.5% | 47.5% | 2.5% | 17.5% | 12.5% | 2.5% |
| Label = 4 | 10.2% | 34.7% | 22.4% | 28.6% | 4.1% | 0.0% | 0.0% |
| Label = 5 | 11.8% | 47.1% | 5.9% | 2.4% | 24.7% | 7.1% | 1.2% |
| Label = 6 | 9.1% | 0.0% | 0.0% | 0.0% | 15.2% | 57.6% | 18.2% |
| Label = 7 | 1.5% | 16.4% | 1.5% | 1.5% | 13.4% | 4.5% | 61.2% |
