# Supplementary figures and images for: A Typology of Social Media Use by Human Service Nonprofits: Mixed Methods Study
Source: J Med Internet Res. 2024 May 8;26:e51698. doi: 10.2196/51698 (PMC11112479; doi:10.2196/51698)

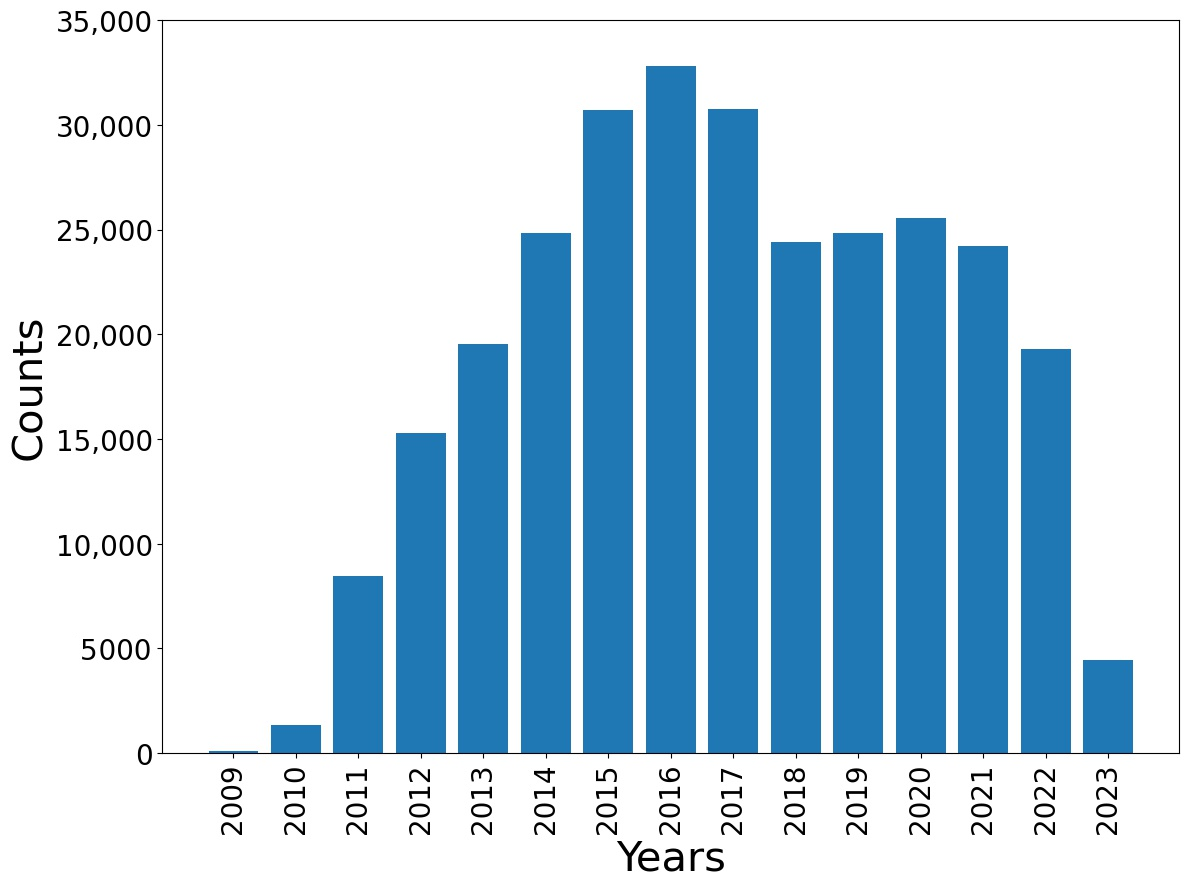

Supplement: Multimedia Appendix 2 [file jmir_v26i1e51698_app2.png]

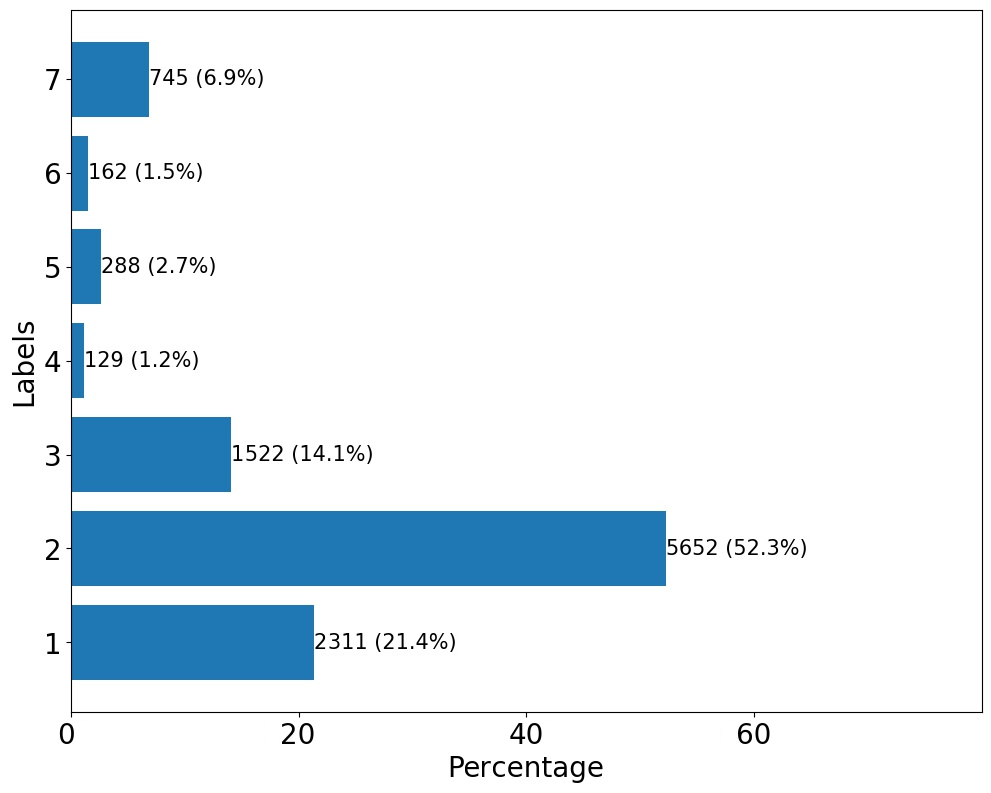

Supplement: Multimedia Appendix 3 [file jmir_v26i1e51698_app3.png]

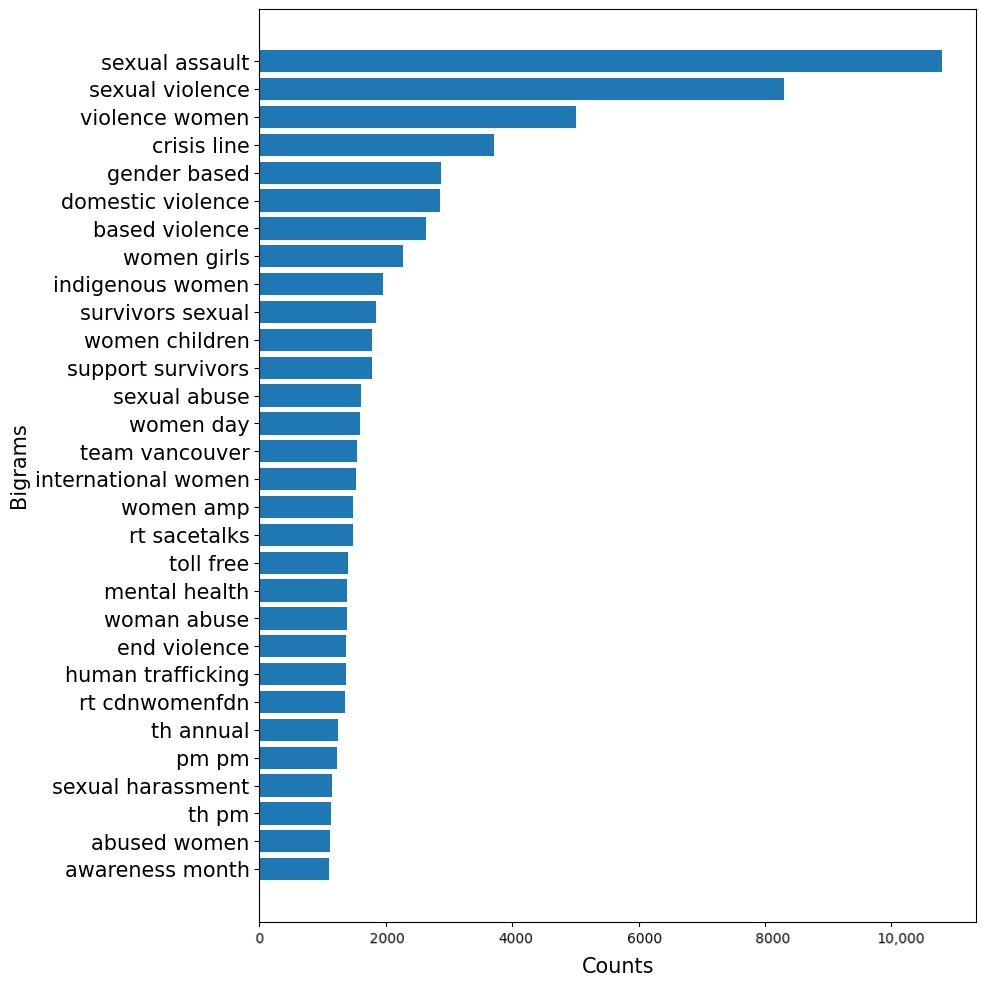

Supplement: Multimedia Appendix 4 [file jmir_v26i1e51698_app4.png]

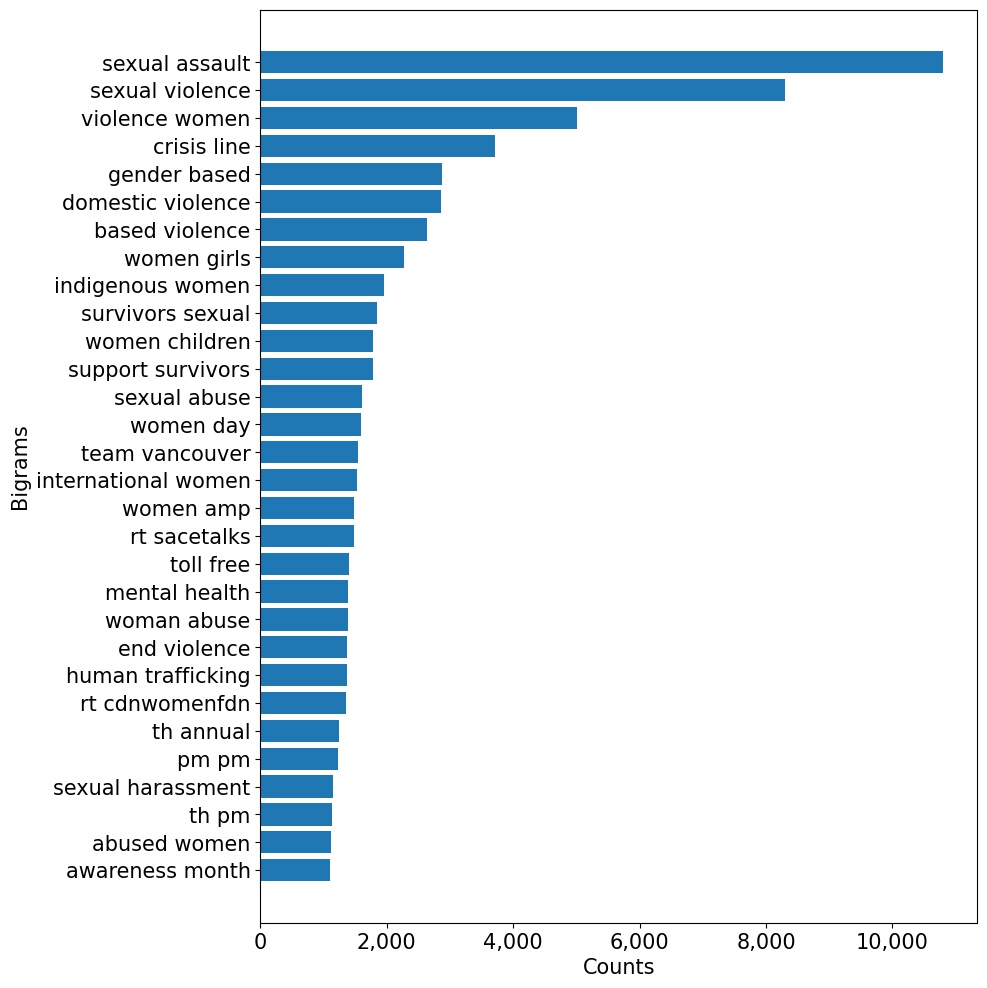

Supplement: Multimedia Appendix 5 [file jmir_v26i1e51698_app5.png]
